# Individual and collective human intelligence in drug design: evaluating the search strategy

**DOI:** 10.1186/s13321-021-00556-6

**Published:** 2021-10-11

**Authors:** Giovanni Cincilla, Simone Masoni, Jascha Blobel

**Affiliations:** Molomics, Barcelona Science Park, c/Baldiri i Reixac 4-12, 08028 Barcelona, Spain

**Keywords:** Collective intelligence, Chemical space exploration, De novo drug design, Artificial intelligence

## Abstract

In recent years, individual and collective human intelligence, defined as the knowledge, skills, reasoning and intuition of individuals and groups, have been used in combination with computer algorithms to solve complex scientific problems. Such approach was successfully used in different research fields such as: structural biology, comparative genomics, macromolecular crystallography and RNA design. Herein we describe an attempt to use a similar approach in small-molecule drug discovery, specifically to drive search strategies of de novo drug design. This is assessed with a case study that consists of a series of public experiments in which participants had to explore the huge chemical space in silico to find predefined compounds by designing molecules and analyzing the score associate with them. Such a process may be seen as an instantaneous surrogate of the classical design-make-test cycles carried out by medicinal chemists during the drug discovery hit to lead phase but not hindered by long synthesis and testing times. We present first findings on (1) assessing human intelligence in chemical space exploration, (2) comparing individual and collective human intelligence performance in this task and (3) contrasting some human and artificial intelligence achievements in de novo drug design.

**Supplementary Information:**

The online version contains supplementary material available at 10.1186/s13321-021-00556-6.

## Introduction

In the last decade, different citizen science initiatives have been promoted to solve complex scientific problems using crowdsourcing and gamification [1–3]. To achieve its objectives, these initiatives make use of individual and collective human intelligence, defined as the knowledge, skills, reasoning and intuition of individuals and groups. Probably the most known projects of this type, developed as on-line video games, are: Foldit, Phylo, CrowdPhase, Udock and EteRNA. Foldit predicts protein structures [4–7] and deals with de novo protein design [8]; Phylo [9] answers multiple sequence alignment questions of comparative genomics; CrowdPhase [10, 11] addresses ab initio phasing issues of macromolecular crystallography; Udock [12, 13] tackles protein–protein docking puzzles and EteRNA [14, 15] solves in vitro RNA design problems. The commonality of these approaches is that they address complex problems with many degrees of freedom where computational approaches struggle to find optimal solutions between the huge number of possible ones.

In the field of small-molecule drug discovery a problem of this type is represented by the drug design process. Actually, designing an ideal drug corresponds to finding an optimal molecule in the chemical space. This is an extremely hard task *inter alia* because the chemical space is huge and finding a specific molecule therein is a needle-in-a-haystack problem.

The chemical space, defined as that abstract entity containing the sum of all drug-like small-molecules, is awfully large. A rigorous method to estimate its extent doesn't exist. The probably most cited size is 10^60^ different molecules, whereas the real number should be somewhere between 10^23^ and 10^180^ [16–22]. What extent of the chemical space has already been explored? To date: 10^8^ molecules have been already synthesized^1^, ^2^; 10^11^ molecules constitute the largest systematic enumeration of all the synthetically accessible molecules up to 17 atoms [23]; and 10^13^ synthetically accessible molecules can be virtually screened [24]. Although reaching such amounts constitutes certainly a great achievement, this is almost insignificant in respect to the total number of possible molecules.

An efficacious way to explore and exploit the chemical space without the need of enumerating huge amounts of molecules is using de novo molecular designers. These are automatic in silico techniques that create molecules from scratch, optimizing certain previously defined requirements (i.e. molecular properties) [25]. Any de novo designer is composed of three elements: a scoring strategy, the method with which molecules are evaluated; an assembly strategy, the approach with which molecules are built; and a search strategy, the technique with which molecules are searched in the chemical space [26]. Many automatic de novo systems have been designed, implemented and tested since almost three decades. They use different scoring strategies (i.e. structure-based [27–29], ligand-based [30, 31]; both coupled with single- and multi-objective optimization approaches [32, 33]), assembly strategies (e.g. atom/bond-based, fragment-based, reaction-based) and search strategies (e.g. Machine Learning [34–39], Genetic Algorithms [30, 40–42]). Although several of these methods have shown promising results, their validation has not been consistent. To solve this problem a suite of benchmarks for de novo molecular design has been recently proposed [43].⁠

The three constitutional elements of de novo designers (i.e. search, assembly and scoring strategies) are not specific of the in silico approach but are general characteristics of the molecule design process. Actually, the same components are part of the classical design-make-test optimization cycles used by medicinal chemists in drug discovery with which initial hit molecules are optimized to leads. Indeed de novo designers carry out virtual design-make-test cycles in silico.

Until today only timid attempts have been made to address drug design using crowdsourcing. Recently some trials were done by integrating many experts in order to: enhance chemical libraries through the “wisdom of crowds” [44], model molecular complexity from a crowdsourced medicinal chemist perspective [45], predict solubility in place of machines [46], and assess quality of molecules generated by automatic algorithms in Turing-inspired tests [47]. All such activities are related to scoring strategies of de novo drug design but no endeavor has been made (as far as we know) to deal with the other two elements: the assembly and the search strategies.

Herein we describe an attempt to use individual and collective human intelligence as search strategies of de novo drug design and quantify their performance. To our knowledge this is the first time that artificial intelligence is substituted by human intelligence in an in silico*, *de novo drug design process. The authors are aware that the term “artificial intelligence” is vague and sometimes misused. However, in this work we have chosen to use this term as a generalization of any computational-based approach that involves a learning activity (e.g. machine learning, genetic algorithms). This was also done to highlight the juxtaposition between human and artificial intelligences, 2 fundamentally different phenomena.

The case study consisted of a series of public experiments addressed to the scientific community where each participant had to explore the chemical space both individually and collectively. From a practical point of view, each participant had to draw and modify molecular structures in a web application in order to maximize a score. Thereby, they had to start the chemical space exploration from scratch, meaning from a single carbon atom, which could be extended and modified to nearly any molecular structure. Each change of structure resulted in a new score. Drawn molecules were saved with their score and could be selected for further modifications by the users. The final objective for participants was to maximize the score.

Participants of this case study engaged in 2 types of experiments: individual and collective ones. The main difference between these 2 experiment types is that while in the individual experiment a participant can access and modify only her/his molecules, in the collective one she/he can do so with the molecules of all participants, making the search collective.

As the first study or its kind, we used a molecular similarity function as a score for the chemical space exploration. This is a typical first step before using more complex, multi-objective functions (e.g. constituted by different machine learning models) that are more suitable for drug discovery programs. In fact molecular similarity is a surrogate for machine learning models and has two big advantages: on one side it is easily interpretable; and on the other side the successful design of the predefined target molecule, towards which the similarity functions achieve their maximum, can be unequivocally determined.

The final objectives of this case study were:

1. Assess human intelligence in chemical space exploration problems
2. Compare individual vs collective human intelligence performances in molecule design
3. Contrast human intelligence with artificial intelligence results obtained in de novo drug design

## Methods

This section describes the methodology used to design, carry out and analyze this case study. Details about the software application used for the study and its implementation are reported in Additional file 1.

### Experiment settings and circumstances

The case study^3^ consisted of a series of public experiments where each participant should find a specific, predefined target molecule in the chemical space. This was supposed to be done by designing molecules from scratch, following a molecular score that indicates how close the solution is. Participants were invited to engage in two experiments: an individual design and a collective design experiment. In the first one they searched the target molecule individually by competing with other participants, while in the second one they did it collectively by collaborating amongst each other.

The scientific community was invited to take part in this case study through social networks (i.e. Twitter, LinkedIn). A web-based application, which is further described in Additional file 1, was developed for this case study. Before being invited to the experiments, participants were asked to create an account on our application and undertake simple learning steps in the *Sandbox*, the application area where one can learn how to draw, save and access molecules. Participants that fulfilled the *Sandbox* requirements were consecutively invited to an individual and a collective design experiment. The beginning of an experiment was scheduled only once at least 10 participants were available. At least 24 h before the experiment started, the participants were notified by an e-mail system which is described in Additional file 1*.* Different experiments could be launched and run at the same time by randomly selecting participants between those who fulfilled the Sandbox requirements. The duration of each experiment was set to the first occurring event, being either the discovery of the target molecule or a time limit of 2 weeks. None of the participants was involved simultaneously in the two experiments associated to them.

Collective but also individual design experiments were run with groups of people for two main reasons. First, the settings of the two experiment types were supposed to be maintained as similar as possible. Second, in this way participants had access to the experiment common ranking that worked as a motivation factor to drive the molecular search.

From a practical point of view, the main difference between an individual and a collective design experiment is that while in the former a participant has only access to the molecules generated by her/himself, in the latter she/he has access at any moment to all the molecules generated by all the participants of the experiment, dynamically.

### The target molecules

In order to assess the human capacity of exploring the chemical space but also compare it to that of automatic de novo methods, five benchmarks were selected from a recently published benchmark suite [43] for de novo drug design. As explained in "Comparison with automatic de novo designers" section, these benchmarks are based on five target molecules of five different complexity levels. For each of these complexity levels, one individual and one collective design experiment were planned, resulting in a total of 10 experiments.

Nevertheless, using the published target molecules of the five selected benchmarks with humans may bring to potential disputes. First, participants of the experiments may be aware of such benchmarks and the target molecules used therein. Second, using exactly the same target molecule for one individual and one collective design experiment may be questionable, as participants of the first experiment may be in contact with participants of the second and could reveal the identity of the target molecules ahead of time. Third, as the target molecules of such benchmarks are approved drugs, they may be known by participants. To overcome such problems while ensuring the validity of the comparison with the benchmarks, 10 complexity-equivalent molecules were selected from ChEMBL database [48–52] between compounds that didn't reach clinical phases.

The choice of such target molecules was dictated by 2 contrasting and opposite necessities. On one side the target molecules should represent pharmacology-relevant compounds, typical of the biological active space, that have already been synthesized and their utility have been experimentally proven. This would suggest to use approved drugs or at least investigational compounds that have reached the clinical development phase. On the other side the target molecules should be unknown to the participants. This would imply to use completely virtual, non-existing molecules that potentially can be synthetically unaccessible. Our choice represents a trade-off between such contrasting cases. Indeed, selecting synthesized (i.e. non-virtual) compounds, typical of the pre-clinical, biologically active space maximizes the pharmacological relevance of the compounds while minimizing the probability that the participants know them.

To ensure the complexity equivalence between the chosen molecules and those used in original benchmarks, the following parameters were set to be the same: number of heavy atoms, number of aliphatic and aromatic rings, molecular fingerprints cardinality (i.e. the number of bits with a non-zero count in the molecular fingerprints) and the number of molecular fingerprints (i.e. the sum of all the individual fingerprints count). In this way, both a size- and complexity-equivalence were warranted. Target molecule complexity level is defined on the basis of fingerprints cardinality.

The original benchmark molecules and complexity-equivalent ones are shown in Table 1. Possible criticism about the design of the experiments is discussed in Additional file 1. The target molecules structures are provided in SMILES format in Additional file 2.

**Table 1:** Target molecules of the selected benchmarks and their corresponding complexity-equivalent target molecules used in this case study

Complexity level	Complexity features	Benchmark target molecule	Individual experiments target molecule	Collective experiments target molecule
L1	# heavy atoms: 17 # aliphatic rings: 0 # aromatic rings: 1 cardinality: 33 # fingerprints: 45	Albuterol	T8 (CHEMBL460262)	T9 (CHEMBL1159712)
L2	# heavy atoms: 26 # aliphatic rings: 0 # aromatic rings: 3 cardinality: 41 # fingerprints: 71	Celecoxib	T13 (CHEMBL1566732)	T32 (CHEMBL461573)
L3	# heavy atoms: 30 # aliphatic rings: 2 # aromatic rings: 2 cardinality: 51 # fingerprints: 85	Thiothixene	T15 (CHEMBL1352527)	T14 (CHEMBL1259158)
L4	# heavy atoms: 30 # aliphatic rings: 2 # aromatic rings: 2 cardinality: 53 # fingerprints: 87	Aripiprazole	T19 (CHEMBL370628)	T20 (CHEMBL554907)
L5	# heavy atoms: 31 # aliphatic rings: 2 # aromatic rings: 2 cardinality: 54 # fingerprints: 86	Troglitazone	T45 (CHEMBL2098358)	T44 (CHEMBL1529981)

For each complexity level, the common complexity features of the target molecules are reported. “Cardinality” is the number of bits with a non-zero count in the fingerprints of target molecules, while “# fingerprints” is the sum of all individual counts

### Molecular score

Every molecule designed in the system by participants was associated to a single-value molecular score. In all the experiments this score corresponded to the Tanimoto similarity [53] towards its target molecule, linearly normalized in the 0–1000 range. The similarity was calculated using 1024-hashed, count-based, diameter-4, extended connectivity fingerprints (i.e. ECFC4_1024 [54]) as implemented in CDK [55–58] (version 1.5.13). The choice of such fingerprints was motivated by 2 main reasons. First, they represent regularly used, general purpose molecular fingerprints. Second, these fingerprints are the same used by the selected benchmarks. It has to be noted that such information was not shared with participants. The only two things they knew about the molecular score were its range and the fact that the higher the score, the closer the target molecule. The same molecular score but not normalized in the 0–1000 range was used for de novo design benchmarks comparison.

### Experiment data, scoring and analysis

Each molecule created in the system may have been drawn starting from scratch or from another molecule already in the system. For each created molecule, the following information was stored *inter alia*: its structure, its score, its creator, its date and time of creation and the molecule from which it derived (if any). With this information it was possible to calculate different parameters to do a complete analysis of the experiments.

- *Maximum score reached* The principal parameter used for the analysis is the maximum score reached in an experiment, represented by the top-1 molecular score calculated as explained in section "Molecular score". The maximum score reached is a measure of the efficacy achieved in an experiment.
- *Number of generated molecules* An interesting parameter for evaluating the efficiency reached in experiments is the number of generated molecules. This corresponds to the number of unique molecules that are generated (and hence tested) to reach the final results. Uniqueness of molecules is calculated on basis of InChIKey, the hashed code derived from the standard InChI [59], the IUPAC International Chemical Identifier.
- *Time played* Another interesting parameter to evaluate the efficiency achieved in experiments is the time played, that is the total time spent by participants in designing molecules. Time played is computed considering the sum of the time frames between all the molecules designed by a participant. To avoid accounting for idle times, frames greater than 1 min were omitted.
- *Scaffold/molecule ratio* It is a parameter that can give information about how focused the molecular search is. This is the ratio between the number of unique molecules and unique scaffolds generated during one experiment. Scaffolds were defined according to Murko's definition [60] as calculated by RDKit.^4^
- *Number of molecule evolution steps* Participants generate molecules in different design sessions, meaning at different moments of time. A design session includes all the molecules that are generated starting from scratch or from a certain molecule already in the system. The number of molecule evolution steps corresponds to the number of different design sessions needed for a certain molecule to be created. This is a particularly important and useful parameter for eventually found target molecules.
- *Collaboration degree* It is defined as the percentage of experiment participants that are involved in the creation of a certain molecule. It is a particularly important and useful parameter for eventually found target molecules.
- *Leader changes* It is the number of times a new leader was recorded during an experiment, representing the events when a new participant overtakes the current highest score and search front.

### Comparison with automatic de novo designers

In order to compare molecule design driven by human intelligence with that guided by artificial intelligence (i.e. de novo designers), this case study was oriented on GuacaMol [43], a recently published benchmark suite for de novo molecular design. There, two types of benchmarks are proposed. First, the distribution-learning benchmarks that evaluate whether a specific method can reproduce the distribution of a certain molecule set. Second, the goal-directed benchmarks that evaluate whether a specific method can generate individual molecules with predefined features (i.e. molecules can be scored individually). The use of GuacaMol goal-directed benchmarks allows to compare the molecular search strategy of humans with that of some recent de novo designers considered state-of-the-art in the field. These systems represent a variety of searching methods as: genetic algorithms (GA) [61], Long-Short Term Memory recurrent neural networks (LSTM) [62] and Monte Carlo Tree Search (MCTS) [63] applied to two molecular representations: graph-based and SMILES-based [64, 65]. In total the following five baseline models are considered in GuacaMol for goal-directed benchmark: *smiles_ga* [66]*,* *graph_ga* [42]*,* *graph_mcts* [42]*,* *smiles_lstm* [38] and *best_of_dataset.* Where: the first four are named after the used molecular representation and the used searching algorithm type, while the fifth is a database virtual screening. This last represents the minimal score and only de novo search strategies that score higher have an advantage over simple virtual screening.

The first five goal-directed benchmarks of GuacaMol were selected, consisting of the three rediscovery and the two similarity benchmarks reported in Table 2.

**Table 2:** Benchmarks selected from GuacaMol [43]

Benchmark name	Benchmark type	Scoring function	Scoring
Celecoxib rediscovery	Rediscovery	sim(Celecoxib, ECFC4)	Top-1
Troglitazone rediscovery	Rediscovery	sim(Troglitazone, ECFC4)	Top-1
Thiothixene rediscovery	Rediscovery	sim(Thiothixene, ECFC4)	Top-1
Aripiprazole similarity	Similarity	Thresholded(0.75) sim(Aripiprazole, ECFC4)	Top-1, top-10, top-100
Albuterol similarity	Similarity	Thresholded(0.75) sim(Albuterol, ECFC4)	Top-1, top-10, top-100

“Scoring” refers to the number of top molecules considered in the score calculation

The aim of a rediscovery benchmark is to evaluate the rediscovery (i.e. re-design) of a single target molecule of interest, while that of a similarity benchmark is to evaluate the generation of many molecules that are closely related to a single target molecule. The scoring function used in the first case is the Tanimoto similarity [53] to the target molecule calculated using ECFC4 fingerprints, while the second one uses the same scoring function adjusted with a 0.75-threshold modifier. As described in the original publication [43], such modifier assigns a full score (i.e. 1.0) to values above a given threshold *t* (in this cases 0.75) while values smaller than *t* decrease linearly to zero. Finally, rediscovery benchmarks base their score on the top-1 molecule generated during the design, while similarity ones on the top-1, top-10, top-100 molecules and their average.

## Results and discussion

### Participation

After the scientific community was called to engage in the case study as described in section "Experiment settings and circumstances", the participation results reported in Table 3 were obtained. A total of 118 participants completed the sign up process; 91 of them accessed the *Sandbox*, where they could learn the basics of the application; 71 completed the *Sandbox* requirements and were invited to the experiments; 46 took finally part in the experiments and 31 of them resulted to be very active, drawing more than 100 molecules each.

**Table 3:** Participation results

Event	Participants
Sign up process completion	118
*Sandbox* access	91
*Sandbox* completion	71
Participation in challenges	46
High activity in challenges (> 100 drawn molecules)	31

46 participants of the initial 118 who signed up (i.e. 39%) engaged in the experiments but 71 out of 91 (78%) who accessed the *Sandbox* could correctly complete its requirements. This means that loss of participants in relation to the possible difficulty of using the application (i.e. 20) represents only 28% of all drop outs, highlighting the ease of participating in the case study. The choice to demand the completion of the *Sandbox* requirements before letting the participants access the challenges allowed them to learn the basics of the application and practice with it without tampering with the data generated in the experiments.

Each of the 71 participants that completed the *Sandbox* requirements was invited to one individual and one collective experiment. The average invitation was 12 participants per experiment while the average engagement (people who draw at least 1 molecule) was 7.

To achieve the highest number of participants, it was opted to keep participant profiling as basic as possible. The participants were asked for the following information: their full name, their e-mail address, and if they studied chemistry/biotechnology/biology or a related discipline so that they feel comfortable in sketching molecular structures (condition for which participants were denoted here as “skilled participants”). Only eight non-skilled participants completed the sign up process, but none of them completed the *Sandbox* necessary to participate in the experiments. In order to achieve similar levels of human knowledge in the individual and collective settings, participants were invited to both experiment types. 83% of people who participated in the collective experiments also participated in the individual ones (see Additional file 1)*.* Finally, it’s worth to mention that no single participant in collective experiments overperformed compared to the others so that the hypothesis that a single participant drove the full collective experiment could be excluded.

The case study successfully recruited dozens of active participants which allowed an acceptable analysis of the observed tendencies and behaviors. Although achieving hundreds or even thousands of active participants would certainly be favorable to obtain more statistically significant results, such ideal situation is very difficult to achieve. A first difficulty is getting into contact and motivating enough skilled participants to enroll in and drive the experiments. In this respect even for very successful scientific games as Foldit, that achieved thousands of sign-ups, most of the puzzles, comparable to our experiments, were basically led by less than 10 people per puzzle (5 people being the median and 6 the mean) [4]. Such few participants were those who improved the experiment score. Another difficulty, triggered to enable collective design dynamics, was that in our case people should participate synchronously in the experiments during at most 2 weeks.

Communication of these first results could, as in case of other scientific on-line games, raise the participation number in future challenges to further support the statistical significance.

### Finding the target molecules

In total, 10 different experiments were conducted to assess human search strategy in chemical space exploration: five individual and five collective ones. Results are reported in Table 4.

**Table 4:** Target and best (i.e. most similar) molecules designed by participants in individual and collective experiments

Complexity level	Individual design		Collective design	
	Target molecule	Best molecule achieved	Target molecule	Best molecule achieved
L1	T8	Score = 1000	T9	Score = 1000
L2	T13	Score = 722	T32	Score = 1000
L3	T15	Score = 605	T14	Score = 1000
L4	T19	Score = 931	T20	Score = 1000
L5	T45	Score = 802	T44	Score = 1000

The first very important result is that in several experiments participants were able to find the target molecule (i.e. score = 1000), that is one specific, predefined molecule among the almost infinite possibilities in the huge chemical space. As far as we know, this is the first time that such a study, quantifying molecule search strategy of humans, is conducted. This result is particularly important considering the following circumstances:

1. Participants searched the chemical space from scratch by drawing molecules starting from a simple carbon atom.
2. As molecules are drawn and manipulated on an atom/bond level, participants had absolute freedom to potentially reach any organic drug-like molecule of the chemical space.
3. Participants searched the chemical space simply by following a single-value molecular score indicating how close they were to the target molecule. They didn't receive any additional hint or information and had to build their own logic behind it.

Target molecules of five different complexity levels were searched. In individual design experiments, participants could only find the most simple target molecule (i.e. T8). Anyway, in the cases of the two most complex targets (i.e. T19 & T45), they got close and reached scores of 931 and 802, corresponding to a Tanimoto molecular similarity of 0.931 and 0.802, respectively. In contrast, in collective design experiments participants could find the target molecule in all the cases.

The scoring function that should be followed in a real drug design program aiming to reach lead compounds would certainly be more complex than the simple similarity function used in such experiments. Indeed it should consider not only the compounds capacity of interacting with the biological target of interest but also their pharmacokinetics (i.e. ADME) and toxicity (T) profile, elements that can be predicted in silico by machine learning models. The choice of using a similarity function in this case study was dictated by two main reasons: (i) it is a surrogate for machine learning models and if a de novo molecular generator doesn't work using similarity functions, probably it will have difficulties in working with more complex functions (this is also why similarity functions are used as basic functions in de novo design benchmarking). (ii) the interpretation of the results is easy and the achievement of the target molecule can be unequivocally determined. Using a similarity function is therefore a useful first step to take before searching more complex scenarios which results have an undoubted intrinsic value.

### Individual vs collective molecule design

Experiment results are reported in Table 5.

**Table 5:** Results obtained by participants in the individual & collective search for specific, predefined target molecules in the chemical space

Target complex. level	Individual design						Collective design							
	Target mol	Time played	Generated unique molecules	Scaffold/molecule ratio	Leader changes	Max score^a^	Target mol	Time played	Generated unique molecules	Scaffold/molecule ratio	Leader changes	Max score	Target mol. evolution steps	Target mol. collaboration degree (%)
L1	T8	6H 24 m	2,402	0.184	6	**1000**	T9	2H 45 m	1,343	0.186	6	**1000**	9	100
L2	T13	16H 13 m	6,821	0.429	11	722	T32	7H 10 m	2,936	0.266	11	**1000**	20	50
L3	T15	9H 53 m	4,544	0.325	9	605	T14	9H 19 m	3,708	0.246	9	**1000**	23	50
L4	T19	27H 42 m	11,660	0.384	7	931	T20	7H 34 m	2,856	0.384	15	**1000**	20	87.5
L5	T45	11H 31 m	5,971	0.381	3	802	T44	19H 40 m	7,842	0.404	13	**1000**	29	100

Target molecules are classified by complexity level. The number of generated unique molecules is reported together with the scaffold/molecule ratio. Leader changes represent the number of times a new leader was recorded during an experiment. The max score is the highest score obtained in an experiment (max = 1000). Target molecule evolution steps is the number of different design sessions required to reach the target molecule. The target molecule collaboration degree is the percentage of experiment participants that are involved in the creation of the target molecule

^a^This is the molecular score visible by the participants in the application. It is different from the scores calculated for the de novo design benchmarks comparison

The following observations can be made on the basis of the results:

1. *Collective design seems more efficacious than individual design* While in the five individual design experiments the target molecule was found only in the simplest case, all the five collective design experiments were successful. This suggests a higher efficacy of collective molecule design in respect to individual one.
2. *Collective design seems more efficient than individual design* Collective design succeeded in finding the target molecule not only by generating (and hence testing) less molecules but also by needing less playing time. There is just one case where the collective design generated more molecules and took more playing time than the individual one: the experiment targeting the most complex target molecule (i.e. complexity level L5). Nevertheless, as the individual search could not find the target molecule, it cannot be concluded that in this case individual design was more efficient.
3. *Collective search is at least as broad as the individual one* One concern about collective design may be that, given a certain number of molecules, it generates less scaffolds in respect to the individual design. This may happen as at any moment in time all participants may center their search around the best molecule (or currently few best molecules) so that fewer scaffolds are generated. This hypothesis seems to be incorrect as it only holds up in two out of five cases, which can be seen on basis of the scaffold-molecule ratio reported in Table 5.
4. *Designing complexity* Interestingly, the number of molecules needed by collective design to reach the target molecule does not correlate with its computationally estimated complexity. Similarly, in case of individual experiments the maximum score achieved does not inversely correlate with the target complexity metrics as it could be expected. This may indicate that the designing complexity experienced by humans differs from the one computationally defined.
5. *Collaboration* The collaboration degree of target molecules in collective design experiments ranges from 50 to 100%, so at least half of the participants of an experiment helped to achieve a target molecule. Similarly, the percentage of participants who created forefront molecules in collective experiments ranges from 83.3% to 100% (see Additional file 1: Table S2). A forefront molecule is the top-1 scored molecule of an experiment at a certain moment in time. This highlights the shared effort of the participants made in collective experiments to search for target molecules. In two of the four experiments where collective design was more efficacious than individual design, more leader changes are observed. Interestingly, the difference is particularly large in case of the two most complex targets (i.e. 15 vs 7 and 13 vs 3 for collective vs individual experiments with target molecule complexity level L4 and L5, respectively). It can be hypothesized that leader changes in collective design is beneficial for reaching the objective.

**Fig. 1:**
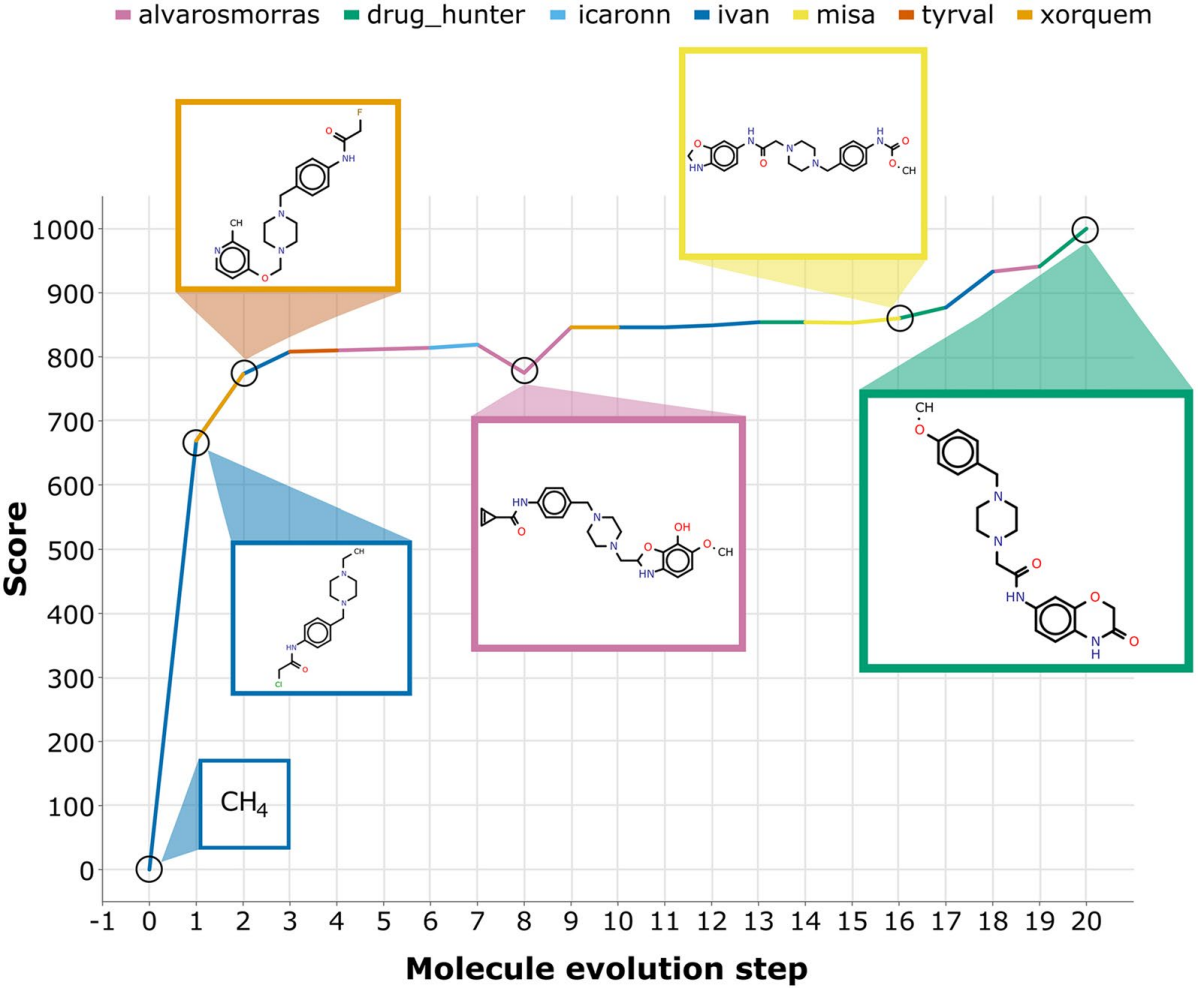
Genesis of target molecule T20. The target molecule is created (i.e. rediscovered) in 20 evolution steps through the collective design efforts of seven out of eight participants of this experiment. The individual contributions to the target molecule creation are represented by different colors. Some intermediate generated molecules are also shown

The evolution steps of the target molecule (defined in Methods) achieved in the five successful collective design experiments ranged from 9 to 29 while their collaboration degree ranged from 50 to 100%. As the possibility to collaborate is the only setting difference between the individual and collective experiments, the high collaboration degree in the creation of the target molecules may be the cause for the higher efficacy achieved in the collective experiments. To illustrate such features, the genesis of target molecule T20 is reported in Fig. 1.

Target molecule T20 was generated in 20 evolution steps through the collective work of seven out of the eight participants of this experiment. While the general trend of molecule evolution is positive, meaning the score of the resulting molecule in each design session is higher than the starting molecule, there are evolution steps in the genesis of target T20 where the score remains equal (steps 10, 11 and 14) or even decreases (steps 8 and 15). The transit through molecules with scores lower than the experiment maximum may represent the exit mechanism from local maxima.

To better understand the differences between individual and collective design, experiments related to complexity-level-L4 target molecules (i.e. T19 & T20) are compared.

**Fig. 2:**
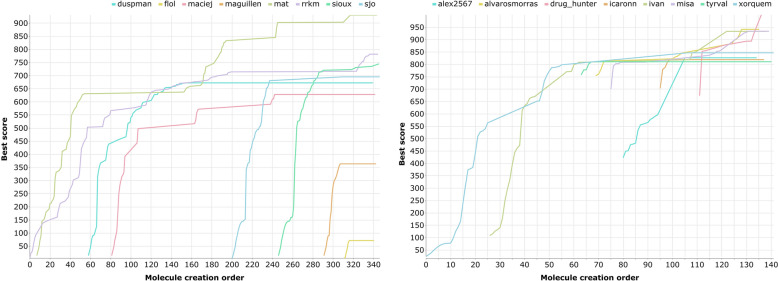
Molecule best scores (y-axis) achieved by participants during individual (left) and collective (right) design experiments of L4-complexity-level target molecule. Molecule creation order (x-axis) is the order in time by which user-based-top-1 molecules were created. A user-based-top-1 molecule is the highest scoring molecule of a single user in an experiment

The top-score achieved by each user along the whole molecule design activity of L4-complexity targets experiments is represented in Fig. 2.

A first consideration is that it seems easier for participants to rise the molecule score from 0 to around 550, than from around 550 to 1000. This is an expected behavior. On one side this may be due to the fact that similarity may rise quickly when some common functional groups are initially added to the structure. On another side, however, this behavior may also reflect a general feature of the chemical space search: it is more difficult to design an optimal molecule (i.e. max score) than a sub-optimal one.

Two main differences emerge from the comparison of the two plots reported in Fig. 2:

- While in the individual design experiment all the participants started the design activity from molecules with a score close to 0, in the collective design one all but the first started exploring the chemical space from already designed molecules with higher scores.
- While the number of leader changes in the individual challenge is limited (i.e. 7), in the collective challenge it is significantly higher (i.e. 15) as everybody can start from the highest scoring molecule.

**Fig. 3:**
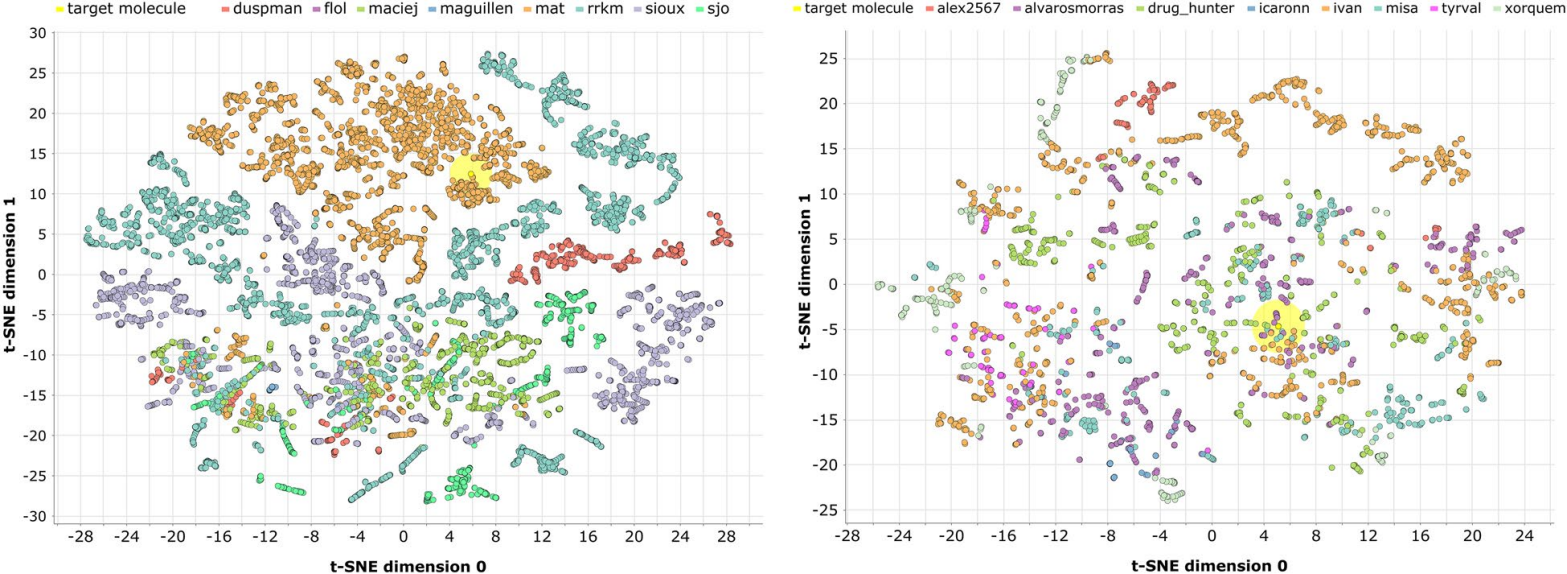
Chemical space explored by each participant during an individual (left) and a collective (right) design experiment. Molecules are described by 1024-hashed ECFC4 fingerprints and represented using a t-SNE visualization. The molecules generated by each participant are represented by a different color. The target molecule is represented by a yellow point highlighted by a halo. The same plot colored by molecule creation order, instead of by participant, is reported as figure S2

To understand the structural diversity of the molecules generated during a design experiment, their distribution in the chemical space can be examined. For such a purpose, molecules are first characterized using the same descriptors with which the molecular score was calculated (i.e. 1024-hashed ECFC4 fingerprints) and then plotted in Fig. 3 using t-SNE (i.e. t-distributed stochastic neighbor embedding).[67].

The following observations can be made about the chemical space plots:

- While in the individual design experiment it seems that specific participants explored specific, focused parts of the chemical space, in the collective design one the molecules generated by each user are more spread in the chemical space.
- In the individual design experiment only one participant came close to the target molecule, while in the collective design one at least four of them.

### Comparison with automatic de novo designers

As described in section "Comparison with automatic de novo designers" this study was designed to compare the search strategy of humans with automatic de novo designers. For such a purpose a recently published de novo design benchmark [43] was chosen that includes results from different automatic methods. Its usage allows also to dissipate any possible doubt that could have arisen if we would have used internal automatic de novo systems for comparison.

**Fig. 4:**
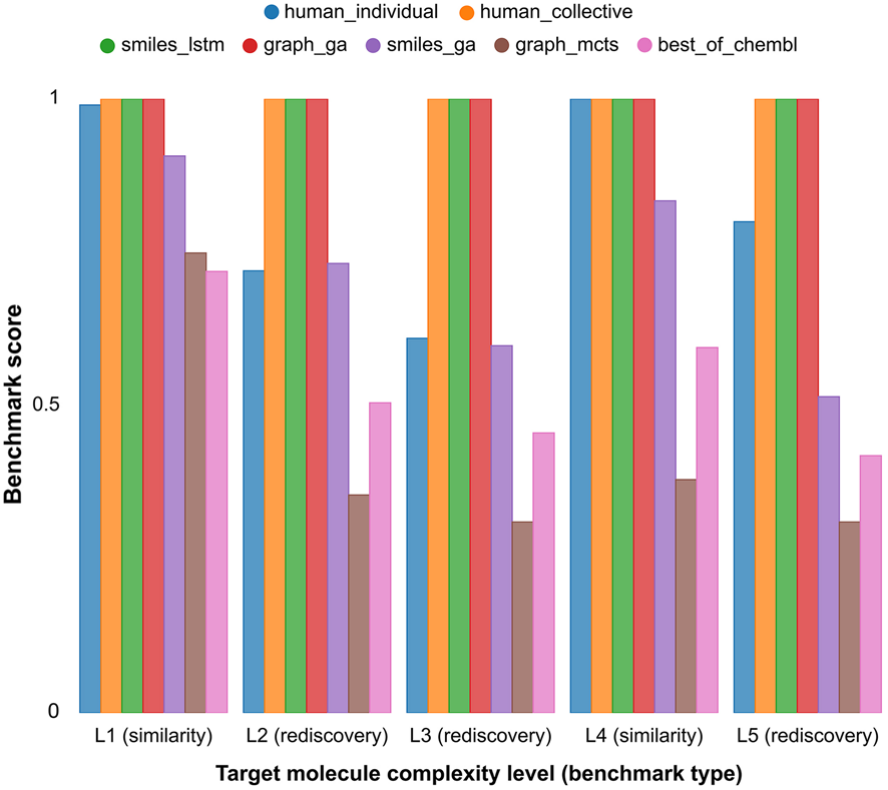
Comparison of human individual and collective design experiments with in silico de novo designers reported in the GuacaMol publication. [43] Benchmark scores are explained in section "Comparison with automatic de novo designers"

The results of both individual and collective human design activity for the five selected benchmarks are reported in Fig. 4 and Table 6 together with those of the state-of-the-art in silico methods published in the original benchmark article [43].

**Table 6:** Comparison of human individual and collective design experiments with automatic de novo designers reported in the GuacaMol publication [43]

Complexity level	Benchmark type	Final score Top-1 score (Top-10 score) (Top-100 score)						
		Human individual	Human collective	smiles_lstm	graph_ga	smiles_ga	graph_mcts	best_of_chembl
L2	Rediscovery	**0.72**	**1.0**	**1.0**	**1.0**	**0.732**	**0.355**	**0.505**
		0.72	1.0	1.0	1.0	0.732	0.355	0.505
L3	Rediscovery	**0.61**	**1.0**	**1.0**	**1.0**	**0.598**	**0.311**	**0.456**
		0.61	1.0	1.0	1.0	0.598	0.311	0.456
L5	Rediscovery	**0.80**	**1.0**	**1.0**	**1.0**	**0.515**	**0.311**	**0.419**
		0.80	1.0	1.0	1.0	0.515	0.311	0.419
L1	Similarity	**0.99**	**1.0**	**1.0**	**1.0**	**0.907**	**0.749**	**0.719**
		1.0	1.0	1.0	1.0	1.0	0.80	0.765
		1.0	1.0	1.0	1.0	1.0	0.758	0.726
		0.96	1.0	1.0	1.0	0.72	0.689	0.664
L4	Similarity	**1.0**	**1.0**	**1.0**	**1.0**	**0.834**	**0.380**	**0.595**
		1.0	1.0	1.0	1.0	0.856	0.428	0.609
		1.0	1.0	1.0	1.0	0.838	0.376	0.601
		1.0	1.0	1.0	1.0	0.807	0.335	0.576

Benchmark scores are explained in section "Comparison with automatic de novo designers". The final score is equivalent to the top-1 score in rediscovery benchmarks and to the average of top-1, top-10 and top-100 scores in the similarity ones

Human collective design performed optimally along all the five tested benchmarks. This is also the case for the two best automatic systems (i.e. smiles_lstm [38] and graph_ga [42]). Human individual design performed more poorly than collective design but still fairly well. Actually, in case of the similarity benchmarks, it achieved almost the optimal scores (i.e. 1.0 and 0.99 in experiments with targets of L4 and L1 complexity level, respectively), while in the case of the rediscovery benchmarks it performed worse than the two best in silico systems, but better than two out of the three other approaches.

In the cases where the benchmark maximum score of 1.0 is not reached, the relation between the complexity of the target molecules and the achieved efficacy is analyzed. Here, efficacy is determined by how close the final achieved score is to the maximum (i.e. 1.0). Interestingly, automatic methods correlate inversely with the estimated complexity levels of the target molecules while this is not true for human individual design. More specifically, this occurs in rediscovery benchmarks (L2, L3 and L5) where smiles_ga = 0.732, 0.598, 0.515, graph_mcts = 0.355, 0.311, 0.311 and human_individual = 0.72, 0.61, 0.80, respectively. This also occurs in similarity benchmarks (L1 and L4) where smiles_ga = 0.907, 0.834; graph_mcts = 0.749, 0.380; human_individual 0.99, 1.0, respectively. While for the automatic methods the molecular design difficulty seems to correlate with the computationally estimated complexity of target molecules, this does not hold up for human design activity.

### Human vs machine learning pace

A possible measure for the learning pace of the search strategy is the number of times the molecular scoring function has been accessed for finding a particular target molecule. The higher the number, the slower the learning pace. In case of human-driven de novo design described herein, this is the number of moves carried out by participants to reach the target molecule. This corresponds to all the (non-unique) molecules generated in the experiments. This number is larger than the number of generated unique molecules reported in Table 5, because it also considers repetitions. In other words, if the same molecule has been drawn five times, it will count as five scoring function calls.

The number of scoring function calls carried out by individual and collective human intelligence are reported in Table 7 together with those of Long-Short Term Memory recurrent neural networks (lstm_smiles) [38], reported^5^ in the GuacaMol [43] publication. Human individual design results are only reported for the experiment where participants reached the target molecule.

**Table 7:** Number of scoring function calls needed to reach the target molecules of five different complexity levels

Complexity level	Number of scoring function calls to reach the target molecule		
	lstm_smiles	Human individual	Human collective
L1	132,838	3614	1956
L2	132,846	–^a^	4271
L3	138,209	–^a^	5404
L4	139,221	–^a^	4591
L5	140,339	–^a^	12,118

Human individual design results are only reported for the experiment where participants reached the target molecule

^a^Target molecule not reached

It can be seen that the number of scoring function calls carried out by humans (in both the individual and collective design mode) are more than one order of magnitude lower than those of the artificial neural network. These results suggest that humans may have a larger learning pace than the considered AI method. The learning pace is related with the efficiency. To determine whether this is a result limited to this case or a general tendency, a larger set of experiments should be carried out and compared to several other machine-based methods. Such an in-depth analysis is beyond the scope of this work.

Interestingly, while the number of scoring function calls needed by artificial intelligence (i.e. lstm_smiles) to reach the target molecule correlates with its complexity level, this does not occur with human intelligence. This observation was also done for efficacy as described above.

This trend should be taken with caution as other AI methods could work differently. The raw data for all the case study experiments is provided in Additional file 3.

## Conclusions

In the last decade individual and collective human intelligence were used in combination with computer algorithms to solve complex scientific problems. These are problems with many degrees of freedom where computational algorithms alone struggle to find the best solution. This approach was successfully used in different research fields as comparative genomics, structural biology, macromolecular crystallography and RNA design. Here we described an attempt to use a similar approach in small-molecule drug design. More specifically we assessed the human search strategy in chemical space exploration problems where specific, predetermined molecules had to be found between the almost infinite possibilities. Finally, results were compared to those obtained by different automatic de novo designers assessed in a recently published benchmark suite. This allows to have a first direct comparison between human and artificial intelligence in de novo drug design.

The here explained case study focused on the usage of a similarity function as design scoring. Although this is certainly a simplification in respect to a drug discovery scenario where more complex multi-objective scoring functions should be used, the molecular similarity is a surrogate for machine learning models and have the advantage of producing easily interpretable results where the achievement of predefined target molecules can be unequivocally determined. In this respect, this study should be regarded as a first necessary step towards the usage of the same approach with more complex scoring functions.

From the results, the following conclusions can be drawn:

1. The search strategy linked to human intelligence can be successfully used in chemical space exploration in silico. Indeed, it is able to find unique, predefined target molecules, having a molecular complexity equivalent to that of approved drugs, between the huge amount of possibilities. This supports the usage of human search capability coupled to in silico molecule evaluation systems in drug design.
2. Collective human molecular design seems to be both more efficacious and more efficient than individual molecular design. This supports the development of collaborative drug design tools that allow to create synergies between different players of this field and reach better drugs.
3. Compared to artificial intelligence systems, the search efficacy of human collective intelligence seems to be at least as good as the best artificial intelligence approaches. In contrast, human individual intelligence ranks average. Considering the search efficiency, these first results suggest that human intelligence may have a higher learning pace than artificial intelligence. Nevertheless, this observation needs to be further explored and validated with additional experiments and their comparison to a larger number of AI systems. Such an in-depth analysis is beyond the scope of this work.

Additionally, some results may suggest that human intelligence perceives molecular complexity differently than artificial intelligence but also in this case more experiments will be needed to confirm such finding. If confirmed, this would support a combined use of the two intelligences in order to reach better drugs. An example of the combined use of these 2 intelligences would be an integrated molecular de novo designer where, given a certain complex scoring function (i.e. different from molecular similarity), the evolution of molecules is guided by a hybrid human-artificial search strategy. Each of the 2 components of such system, meaning humans and machines, can learn and take advantage from the molecular proposals of the other. This can lead to new, structurally diverse, in silico-optimized molecules not otherwise achievable. Summarizing, we expect that the regions of the chemical space reachable with such an hybrid system (and the potential optimal molecule therein contained) are not reachable by non-hybrid approaches guided by each of the 2 “pure” search strategies. In our group we are currently working on two main topics: the extension of the current study with more complex, multi-objective scoring functions; and the implementation of the just mentioned hybrid de novo designer.

## Supplementary Information

**Additional file 1.** Supplementary information file 1.

**Additional file 2.** csv file containing target molecule structures in SMILES format.

**Additional file 3.** raw data of case study experiments.

## Data Availability

All data generated and analyzed during this study are included in this published article and its supplementary information files. Participants' data has been anonymized.

## Footnotes

1 As 26/03/2021 PubChem contains 109,816,593 compounds. https://www.ncbi.nlm.nih.gov/pccompound?term=all%5Bfilt%5D&cmd=search.

2 As 26/03/2021 CAS registry contains more than 180 million unique organic and inorganic chemical substances (https://www.cas.org/support/documentation/chemical-substances).

3 http://molomics.com/explore.

4 RDKit: Open-source cheminformatics. http://www.rdkit.org.

5 This is the only method for which a reliable number of scoring function calls is reported in the original publication (private communication with authors).
