# Supplementary material for: Individual and collective human intelligence in drug design: evaluating the search strategy
Source: J Cheminform. 2021 Oct 11;13:80. doi: 10.1186/s13321-021-00556-6 (PMC8507178; doi:10.1186/s13321-021-00556-6)
Supplement: Supplementary file 1 — Additional file 1. Supplementary information file 1. [file 13321_2021_556_MOESM1_ESM.pdf]

Supporting information

for

Individual & collective human intelligence in drug design: evaluating the search strategy

Characteristics of the application

In order to exploit human molecular search strategy and especially to trigger a fruitful collaboration between participants, the following requirements had to be fulfilled by the software application:

- **Easy to access**. To maximize the access avoiding installation and configuration problems that participants can face, the software was implemented as a web-application accessible by the most common Internet browsers (i.e. Mozilla Firefox, Google Chrome, Apple Safari and Microsoft Edge). This also enabled the possibility for the application to be used in several devices maximizing its potential use.
- **Easy to use**. The application was designed for easy and quick usage. Several different features had been implemented to achieve this. They will be mentioned below in the application components description.
- **Real-time responsiveness**. To take full advantage of the human chemical search strategy, it's essential that participants receive a real-time feedback upon their actions. This means that for any modification they propose on a molecular structure they can receive an instantaneous feedback on that modification. In this way their molecular hypothesis can be quickly checked and their **reasoning and intuition are** combined in a single agile process. Such process can be seen an instantaneous surrogate of classical design-make-test cycles carried out by medicinal chemists during the drug discovery hit to lead phase but not hindered by long synthesis and testing times.
- **Collaborative**. Different collaborative mechanisms were implemented in the application in order to trigger productive cooperation between participants. They will be mentioned below in the application components description.

Design of the application

The application is composed by the four components described below:

- **Chemical space explorer**. Probably the most important component, it was used by participants to create, modify and evaluate molecules. It is constituted by two sub-components: a first-of-its-kind molecular drawer and a molecular evaluator.
  The **molecular drawer** was designed to maximize its ease of use and the speed with which molecules can be created. For this reason it is equipped with: an automatic 2D structure

optimizer, so that molecules are always nicely shown in the way how a chemist would depict them; a structural change prompter, suggesting which modifications can be applied to a molecular structure and where; an automatic aromatizer that allows to aromatize rings with a single click.
The molecular drawer is coupled with a **molecular evaluator** that gives real-time feedback for any modification applied to the molecular structure. The feedback is given by showing the current molecular score and by adding a point in a 2D plot representing the genesis history of the current molecule. Such 2D plot expresses the molecular score surface of the visited chemical space. Additionally, specific molecule pairs of the 2D plot can be compared so that effects produced by modification of functional groups can be easily analyzed.

- **Molecular browser.** Any molecule drawn by any participant is saved and stored on the system back-end, even those that were not meant to be drawn. For example, if for evolving a certain molecule *A* into another molecule *B* a participant has to pass through five other intermediate molecules, all of these are evaluated and saved by the system. In this way, similarly to what happens with synthesis chemistry, where the bioactivity is often tested also for intermediate reaction compounds, intermediate *in silico* molecules are also scored. The molecular browser allows any participant to scan any molecule she/he (in the individual design experiments) or she/he & the team (in the collective design experiments) have created. Upon browsing, molecules can be selected to be modified and evolved. Molecules can be browsed in different ways (e.g. by score range, by creator).
A special mechanism was implemented to summarize at a glance the best molecules generated at any time by all the users of a collective experiment. A reduced set of five molecules defined as "human collective best set" is highlighted. This corresponds to the molecule set that maximizes at once the molecular score and the structural diversity. This was achieved with the score erosion algorithm[1] as implemented in KNIME[2] v.4.1.1.

- **Notification system**. The application is provided with a user-specific notification system, similar to that used in social networks, to update the participants on the progress of the experiments. There are two levels of notifications: instantaneous *in app* notifications and e-mail notifications. The first is thought to inform participants while they are connected to the application, while the second informs them with a daily e-mail when there are unread notifications. Examples of notified events are: change in the experiment leader, change in participants ranking position, etc.

- **Dashboard**. By accessing the application, a dashboard page summarizes at a glance the progress of the experiment. The dashboard shows the following global- and logged-user statistics: the best score achieved, the number of generated unique molecules and the total time played. The participants ranking based on the best score achieved is shown. This is thought to act as a motivation factor for participant engagement. All the data reported in the dashboard are dynamically updated in real-time.

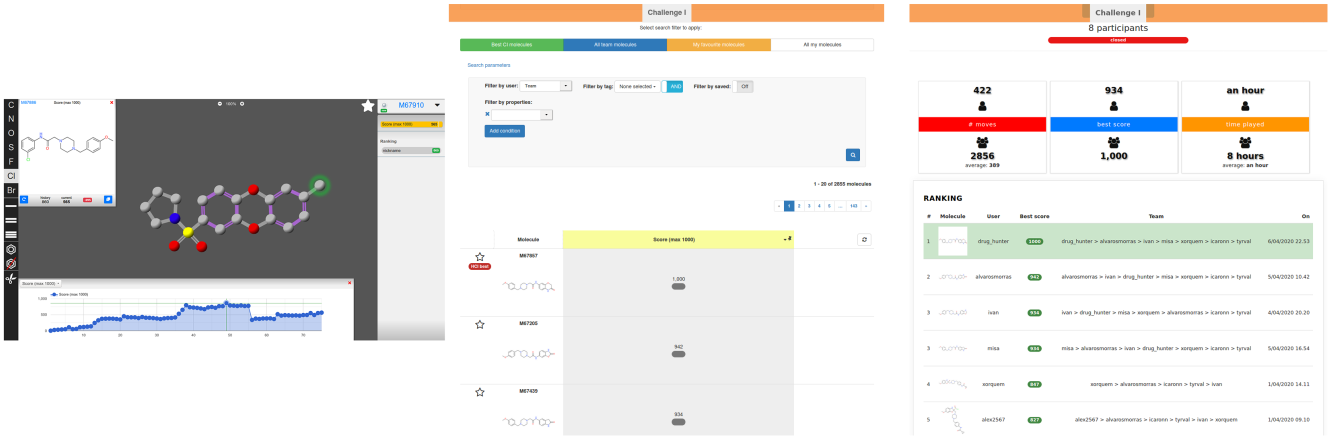

Figure S1: Screenshots of the application. Examples of the chemical space explorer (left), the molecular browser (center) and the dashboard (right).

Example screenshots of the application are reported in Figure S1.

Technical implementation

The web-application developed for the case study is divided in two parts: front-end and back-end.

The front-end was developed using **Angular**[a] for pages and navigation, while the chemical space explorer was developed using **Phaser,**[b] a javascript framework used to develop video games.

The back-end was developed in **Java** using a reactive approach through **Vert.x** tool-kit.[c] The communication between front-end and back-end was implemented using **websocket protocol**.[d] This technical choice made it possible to meet the real-time responsive requirements.

Data was stored in a clustered instance of **MongoDB**,[e] a NoSQL database, giving a low latency and high throughput solution to manage the large amount of data generated by the application.

The whole application was packaged in a **Docker** container[f] and installed in the **AWS** cloud[g]. Through this distributed environment every participant could reach the application in an efficient way.

The application as a *de novo* designer

The application used in this case study can be seen as a human-driven *de novo* designer. While its scoring and assembly strategy are guided by machines, its search strategy is driven by humans.

The scoring strategy of a *de novo* designer is the component responsible to evaluate the generated molecules and to guide the search in the chemical space. Although potentially any kind of scoring function able to relate a molecular structure to a numerical value can be associated with the system

---

a https://angular.io/
b https://phaser.io/
c https://vertx.io/
d https://en.wikipedia.org/wiki/WebSocket
e https://www.mongodb.com/
f https://www.docker.com/
g https://aws.amazon.com/

described herein, the one used in this case study is a molecular similarity. Molecular similarity was chosen as it is a surrogate for machine learning models and it can be used to find unique, specific and predefined molecules in the chemical space (i.e. constituting a predetermined solution to the problem), as also proposed by GuacaMol.[3] Moreover molecular similarity has two big advantages: on one side it is easily interpretable; and on the other side the successful design of the predefined target molecule, towards which the similarity functions achieve their maximum, can be unequivocally determined.

The assembly strategy of a *de novo* designer determines how molecules are built. Generally speaking, an assembly strategy tries to achieve a balance between two desirable but contrasting features. On one side it would be desirable that the assembly strategy would be as free as possible so that any molecule in the chemical space can potentially be created so that the optimal molecules can be found. On the other side, the more freedom is given to the assembly strategy, the more probable that proposed molecules are synthetically not accessible or chemically not stable. In this second case, although the *in silico* score of such molecules is good, they are completely useless. As this case study wants to assess the efficacy & efficiency of human intelligence in reaching target molecules in the chemical space, the assembly strategy freedom associated to this *de novo* designer is almost absolute. In fact, the application allows to generate and modify molecules on an atom/bond level through a molecular drawer so that potentially any molecule of the chemical space can be reached. The only applied restriction is that only valency-correct molecules can be drawn starting from the following heavy atom types: C, N, O, S, F, Cl, Br.

The search strategy of a *de novo* designer is the component that, considering the score of the already generated molecules, leads the chemical space search towards the most promising and productive areas. In the here described application the search strategy cannot be properly outlined *a priori* as it is completely defined by human knowledge, skills, reasoning and intuition.

Experiment design criticism

One possible criticism of the experiment design is related to the public availability of chosen target molecules, present in the ChEMBL database. Although participants did not know this and neither the nature of the molecular score, they could have performed similarity searches of case study high scoring molecules with ChEMBL compounds to find the target molecules. Nevertheless, if this would have happened, the search for the target molecule would be characterized by 2 clearly distinct dynamics. The first part of the search would have been carried out using human reasoning as expected. The second and last part of the search would have been done by simply drawing the found ChEMBL molecules in the application. In this sense, the second part of the search would have been characterized by:

1. The participation of just 1 person
2. A much faster molecule drawing speed (because it wouldn’t require reasoning)
3. A low number of drawn (i.e. tried) molecules

A reasonable similarity value with which molecules could be found in ChEMBL is with a $0.8 \leq$ similarity < 0.85. For each experiment we analyze the data of the target molecule design starting from such similarity range. Data is reported in table S1.

Target complexity level	Target molecule	Design type	Experiment inter-molecule average time ± SD (s)	Required to design the target molecule starting from a similar predecessor molecule (0.8 ≤ similarity < 0.85)			
				Inter-molecule average time ± SD (s)	# unique molecules	# of participants	# of design sessions
L1 (ind)	T8	Individual	5.5±6.1	10.0±9.0	28	1	2
L1 (coll)	T9	Collective	5.0±6.2[h]	5.4±8.4[h]	142[h]	1[h]	2[h]
L2	T32	Collective	6.2±6.8	6.3±8.3	89	2	13
L3	T14	Collective	6.1±6.0[h]	5.2±3.5[h]	84[h]	2[h]	4[h]
L4	T20	Collective	5.6±6.5	6.0±7.5	768	5	30
L5	T44	Collective	6.1±6.0	5.3±5.0	699	3	23

Table S1: Design of target molecules starting from similar predecessor molecule with a 0.8 ≤ similarity < 0.85). Inter-molecule average time and its standard deviation (SD) is the time required by participants to draw a molecule.

From the data reported in Table S1, no significant acceleration in the drawing of molecule starting from the predecessor molecule with 0.8 ≤ similarity < 0.85 can be observed. On the contrary in 4 out of 6 cases the molecule drawing process slightly decelerated. In respect to the number of unique molecules drawn when starting from the predecessor molecule, the number of molecule is sufficiently high in all but one case (i.e. T8) to exclude the hypothesis that molecules are copied from ChEMBL. In case of T8, the low number of unique molecules needed to reach the target could certainly be due to its low complexity (i.e. L1). Moreover, the inter-molecule average time significantly increased when starting from the predecessor molecule with 0.8 ≤ similarity < 0.85, supporting a regular experiment progress for T8 (where no ChEMBL searches were done).

Forefront molecule design

A forefront molecule is a molecule that at a certain moment in time has been the top-1 scored molecule of an experiment. These molecules lead the search. Here we report, for each experiment, the percentage of participants who designed forefront molecules. In case of collective experiments these were the compounds that effectively contributed to the search for target molecules. In case of individual experiments forefront molecules were visible only by their creator.

---

h This data is relative to a predecessor molecule with a 0.85 ≤ similarity < 0.90 instead of a 0.80 ≤ similarity < 0.85. In this case the search for the target molecule didn’t start from any molecule with a 0.80 ≤ similarity < 0.85.

Target complexity level	Individual design			Collective design		
	Target molecule	# of created forefront molecules	Percentage of participants forefront molecules creators	Target molecule	# of created forefront molecules	Percentage of participants forefront molecules creators
L1	T8	46	55.6	T9	36	100
L2	T13	36	50.0	T32	39	100
L3	T15	40	75.0	T14	56	83.3
L4	T19	71	37.5	T20	61	87.5
L5	T45	44	33.3	T44	103	100

Table S2: Forefront molecule creators.

From the data it can be seen that the percentage of participants who created forefront molecules is significantly higher in the collective experiments in respect to the individual ones. Additionally the very high percentages of the collective experiments highlight the shared effort of the participants in the search for target molecules.

Chemical space exploration alternative plot

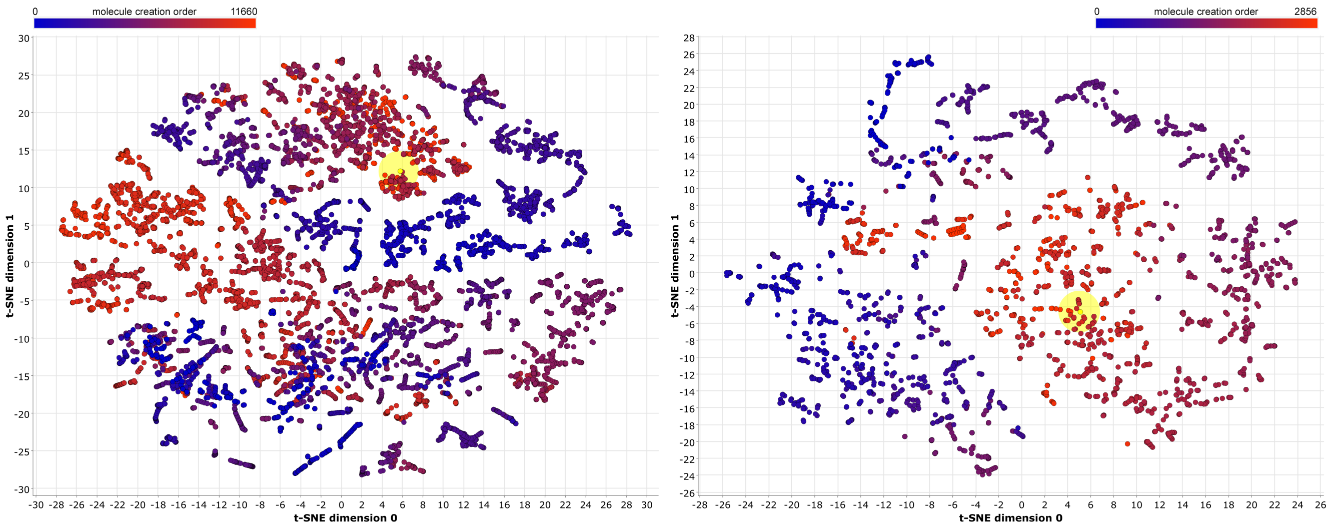

Figure S2 Alternative version of Figure 3 of the article showing the chemical space explored by each participant during an individual (left) and a collective (right) design experiment. Here the plot is colored by molecule creation order, instead of by participant. Molecules created later in the collective experiment tend to focus around the target molecule area.

Participants

Table S3 contains the list of people who participated in the case study experiments. Only people who

accepted to be mentioned are reported.

Alberto Massarotti
Alexandre Borrel
Àlvaro Serrano
Andrew Palmer
Antoine Taly
Benedict Irwin
Benjamin Thornton
Brice Hoffmann
Chase Smith
Danah AlShaer
Edwin Tse
Elisabet Teixido
Fabio Surfaro
Florent Langenfeld
Hadra Sioux Banks Machado
Ivan Rodriguez Torrecillas
James Wagstaff
Jan Jensen
Jesus Garcia
Jonathan Gabriel Piccirillo
Jordi de Mier Vinue
Kévin Launay
Laia Josa Culleré
Laurianne David
Maciej Majewski
Marian Guillen
Mateusz Biesaga
Mireia Rosell
Míriam Martínez-Cartró
Nicanor Zalba
Ognjen Perisic
Oliver Cottrell
Rémi Plainard

Roser Borras Tuduri
Serena Piticchio
Sergio Royuela
Sunhwan Jo
Tania Pereira
Valentin Thirion
Viki Kumar Prasad

Table S3: List of people who participated in the case study experiments. Only people who accepted to be mentioned are reported.

Table S4 contains the list of people who helped in the early testing and tuning of the application with which the case study was carried out. Only people who accepted to be mentioned are reported.

Antonio Pineda Lucena
Borja Mateos
Erwan Guillotel
Estefania Rusca
Ian Craig
Joan Coines
João M. C. Teixeira
Josephine Alba
Marco Chienna
Natàlia Llopart Jiménez
Nicolas Bosc
Ramon Crehuet

Table S4: List of people who helped in the early testing and tuning of the application with which the case study was carried out. Only people who accepted to be mentioned are reported.

Bibliography

(1) Meinl, T.; Ostermann, C.; Berthold, M. R. Maximum-Score Diversity Selection for Early Drug Discovery. *J. Chem. Inf. Model.* **2011**, *51*, 237–247.

(2) Berthold, M. R.; Cebron, N.; Dill, F.; Di Fatta, G.; Gabriel, T. R.; Georg, F.; Meinl, T.; Ohl, P.; Sieb, C.; Wiswedel, B. KNIME: The Konstanz Information Miner. In *4th International Industrial Simulation Conference 2006, ISC 2006*; Springer, 2006; Vol. 11, pp. 26–31.

(3) Brown, N.; Fiscato, M.; Segler, M. H. S.; Vaucher, A. C. GuacaMol: Benchmarking Models for De Novo Molecular Design. *J. Chem. Inf. Model.* **2019**, *59*, 1096–1108.
